# Supplementary material for: Rational design of a robust aluminum metal-organic framework for multi-purpose water-sorption-driven heat allocations
Source: Nat Commun. 2020 Oct 9;11:5112. doi: 10.1038/s41467-020-18968-7 (PMC7547100; doi:10.1038/s41467-020-18968-7)
Supplement: Supplementary file 1 — Supplementary Information [file 41467_2020_18968_MOESM1_ESM.pdf]

Supplementary Information

Rational design of a robust aluminum metal-organic framework for multi-purpose water-sorption-driven heat allocations

Cho *et al.*

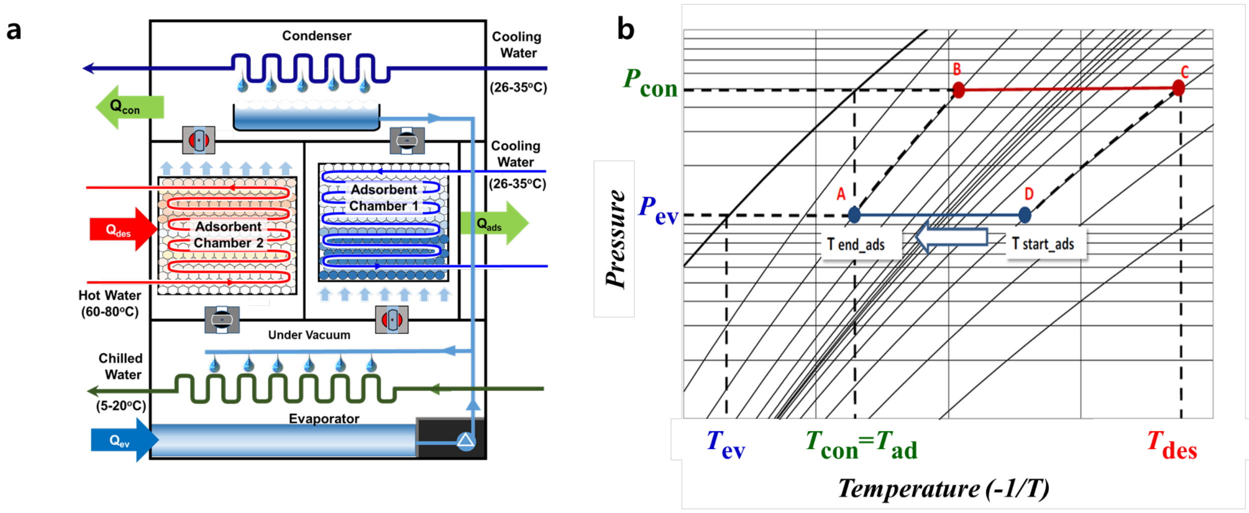

Supplementary Figure 1. a, Typical operation of adsorptive heat transformation (AHT) cycle consisting of adsorption stage (chamber 1) and desorption stage (chamber 2)[1,2]. The arrow direction shows the heat transfer while adsorbing or desorbing the water. Low temperature heat is depicted in blue, medium temperature heat in green, and high temperature heat in red. Notation: heat of evaporation Q_{ev}, heat of adsorption Q_{ads}, heat of desorption (or regeneration) Q_{des}, and heat of condensation Q_{con}. **b**, Typical adsorptive cooling cycle plotted in the P-T diagram[3]. Notation: temperature and pressure of the evaporator (T_{ev}, P_{ev}) and the condenser (T_{con}, P_{con}) and desorption temperature (T_{des}).

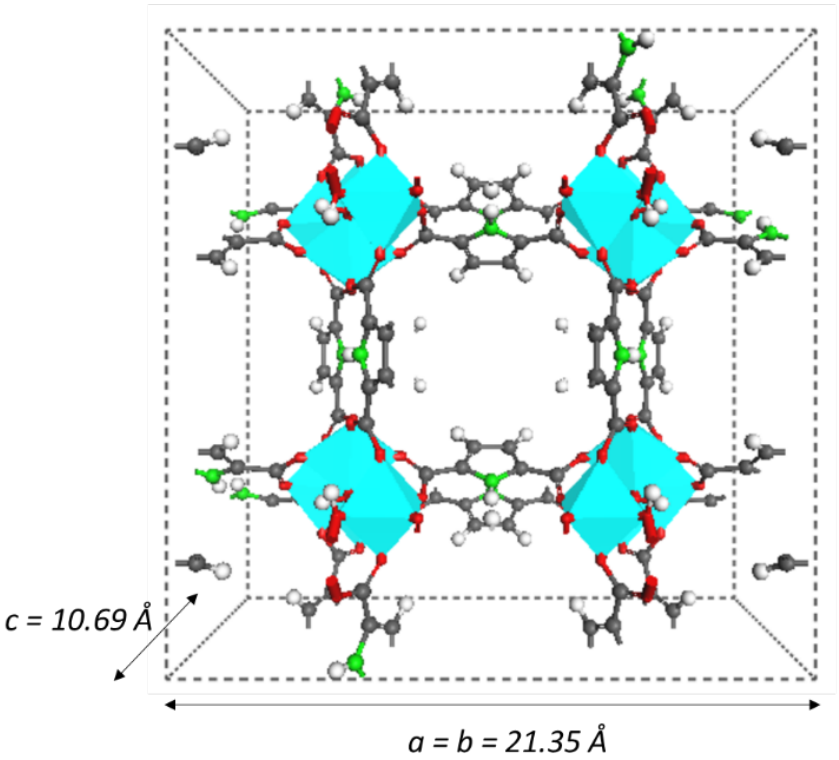

Supplementary Figure 2. DFT optimized structure of the *in silico* constructed structure model for KMF-1.

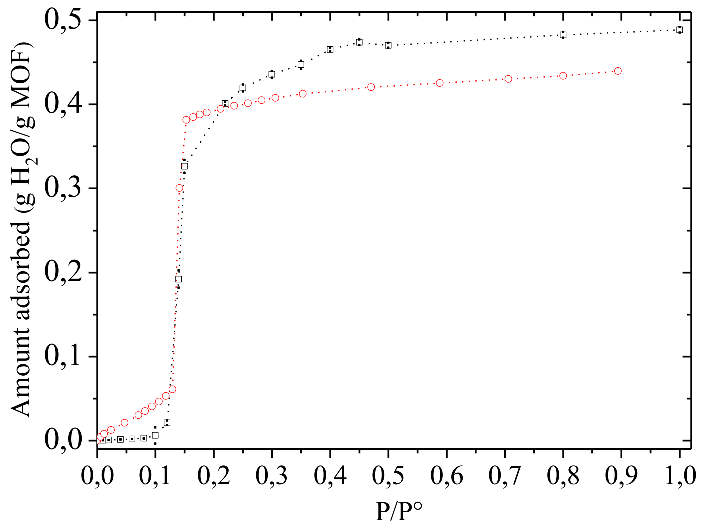

Supplementary Figure 3. Comparison between the GCMC predicted (black) and experimental (red) water adsorption isotherms for KFM-1 at 30 °C. Error bars are included for the simulations.

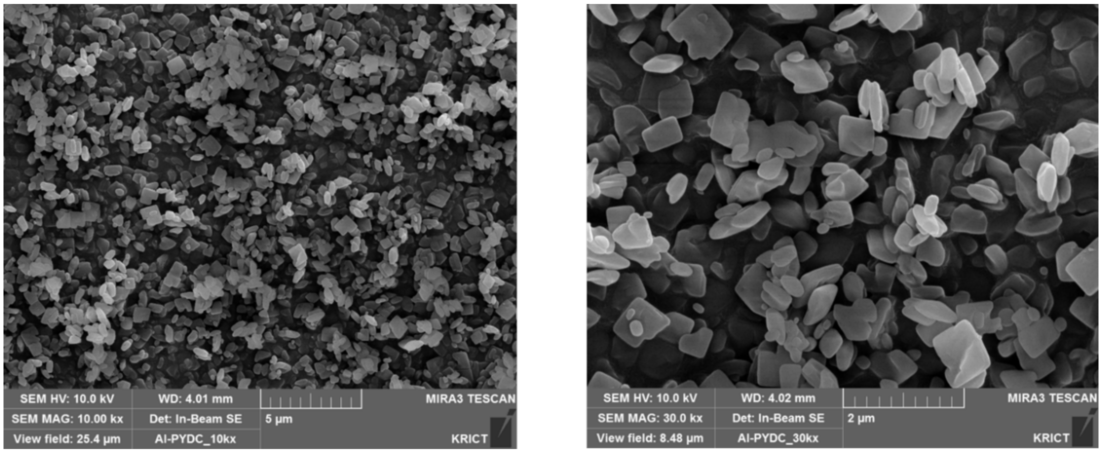

Supplementary Figure 4. SEM images of pristine KMF-1.

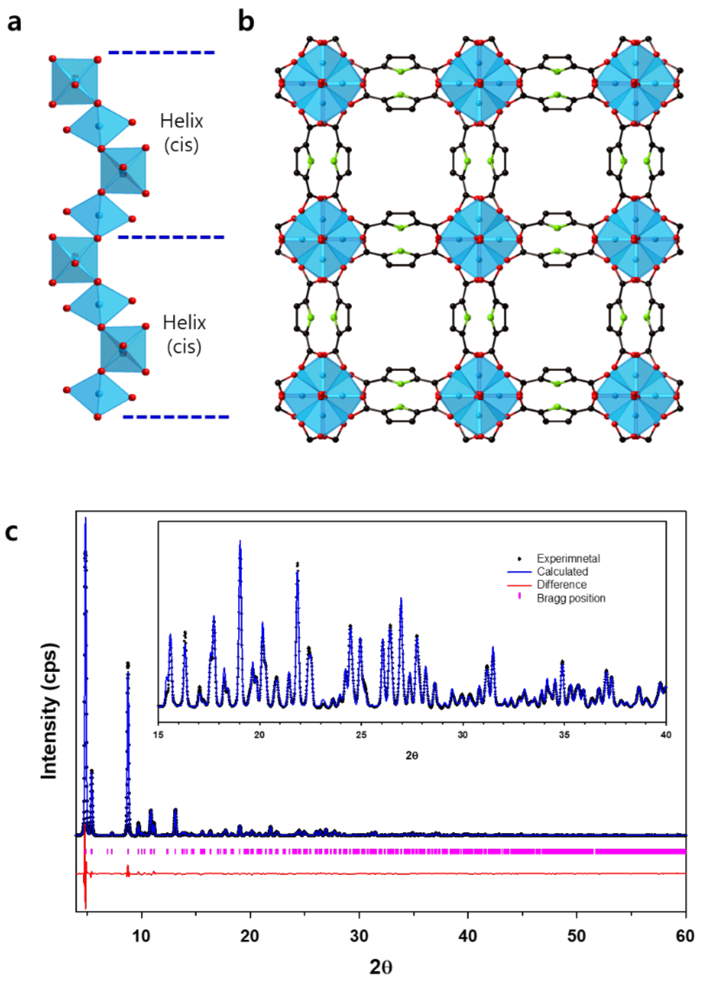

Supplementary Figure 5. Crystal structure determination of the anhydrous KMF-1. **a**, helical chains cis-connected Al octahedra by corner and coordinated by four carboxylates from ligand along a-axis. **b**, Crystal structure of anhydrous KMF-1; **c**, Rietveld refinement of the anhydrous KMF-1 structure model. The measurement is shown in black, simulation in blue and difference in red below. Allowed Bragg reflection are indicated as magenta ticks below. (hydrogen atoms omitted for clarity). Color scheme: Al, blue; C, black; O, red; N, green (hydrogen atoms omitted for clarity).

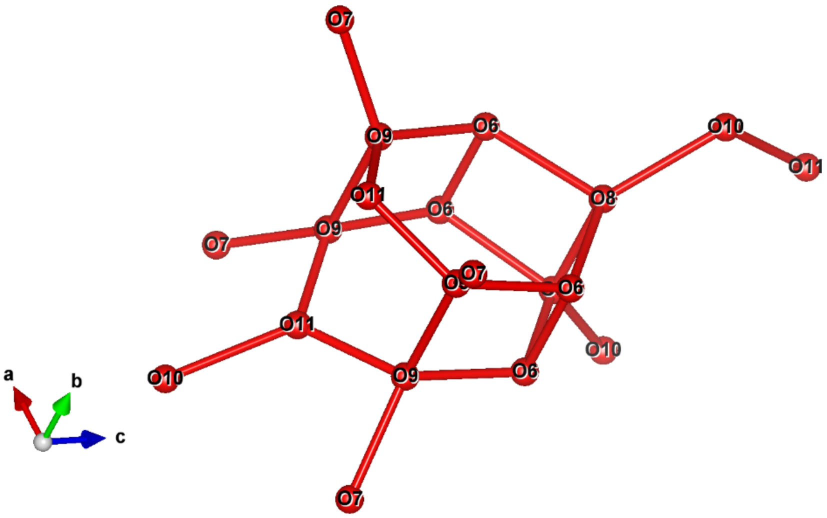

Supplementary Figure 6. Water cluster model for the hydrated KMF-1. Guest waters in the channel of KMF-1 are shown, in which the oxygen-oxygen distance was in the range of 2.56-2.97 Å. The bond connectivity was constrained from 2.5 to 3.0 Å. The framework of KMF-1 was removed for clarity.

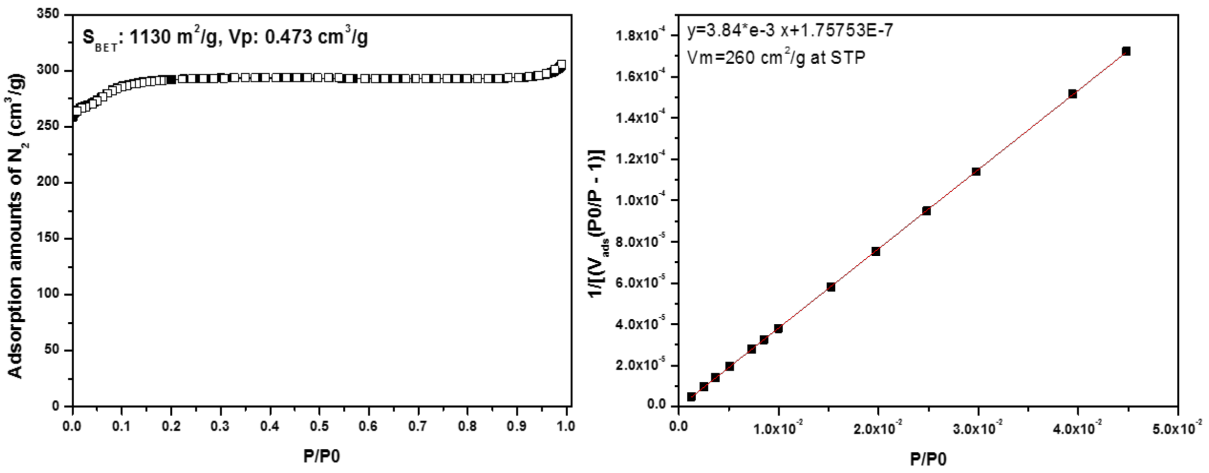

Supplementary Figure 7. Nitrogen physisorption isotherms for KMF-1 measured at -196 °C after pretreated at 150 °C for 12 h (*Left*) and A plot of $V_{ads}(1-P/P^o)$ vs P/P^o for determining the linearity toward BET equation (*Right*) and corresponding BET area based on the fitted red line.

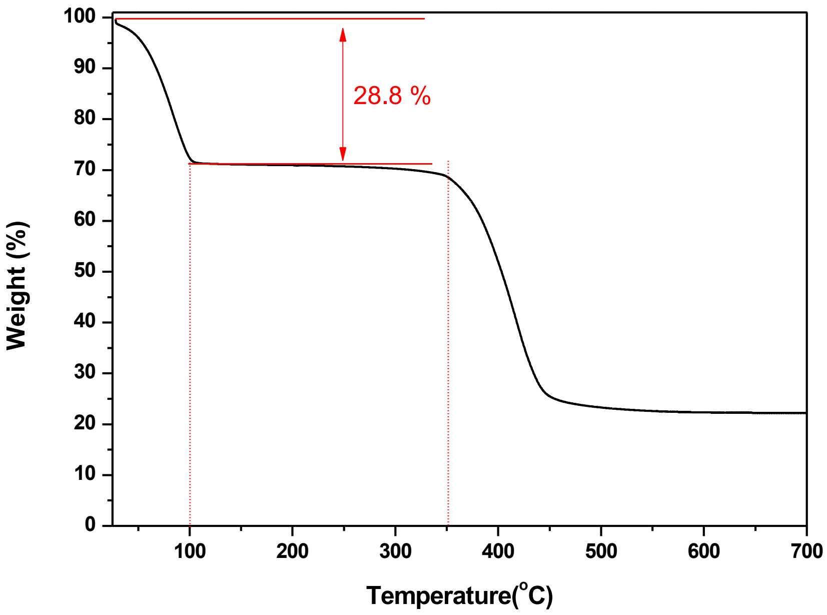

Supplementary Figure 8. Thermogravimetry analysis of KMF-1 after saturated with water vapour under 80 % of relative humidity. The data was collected under 10 °C min^{-1} of heating rate and 30 ml min^{-1} nitrogen flow. 28.8 % weight loss less than 100 °C is desorption weight fraction of pre-adsorbed water.

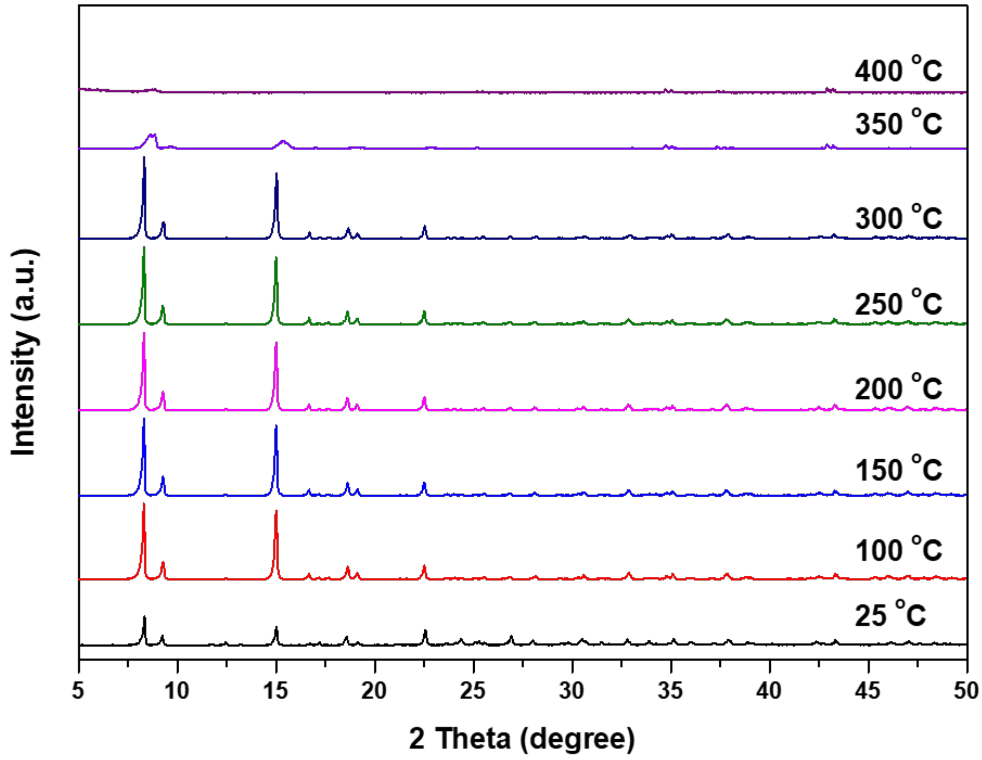

Supplementary Figure 9. PXRD patterns of KMF-1 collected by increasing temperature from 25 °C to 400 °C.

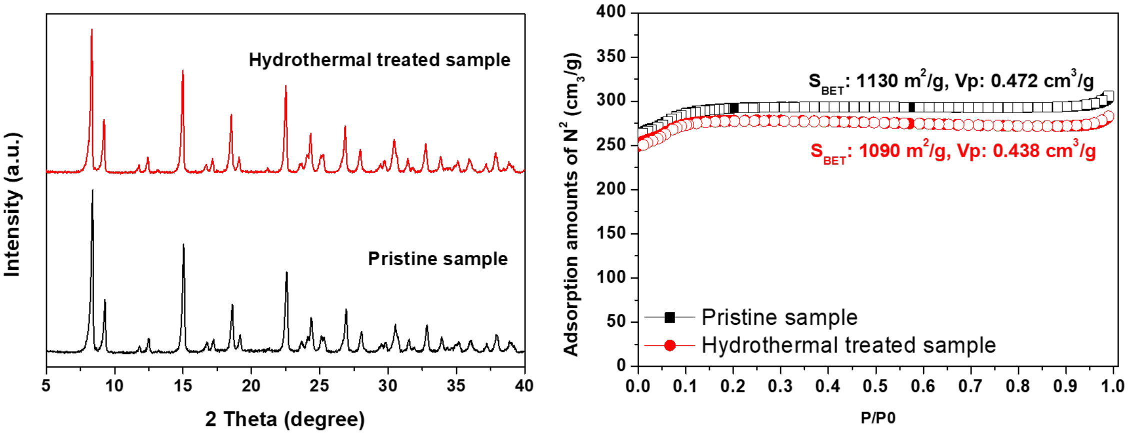

Supplementary Figure 10. PXRD patterns (*left*) and nitrogen physisorption isotherms at -196 °C (*right*) for KMF-1 before and after hydrothermal treatment. The hydrothermal treatment was carried out using boiling water at 100 °C for 24 h under static condition.

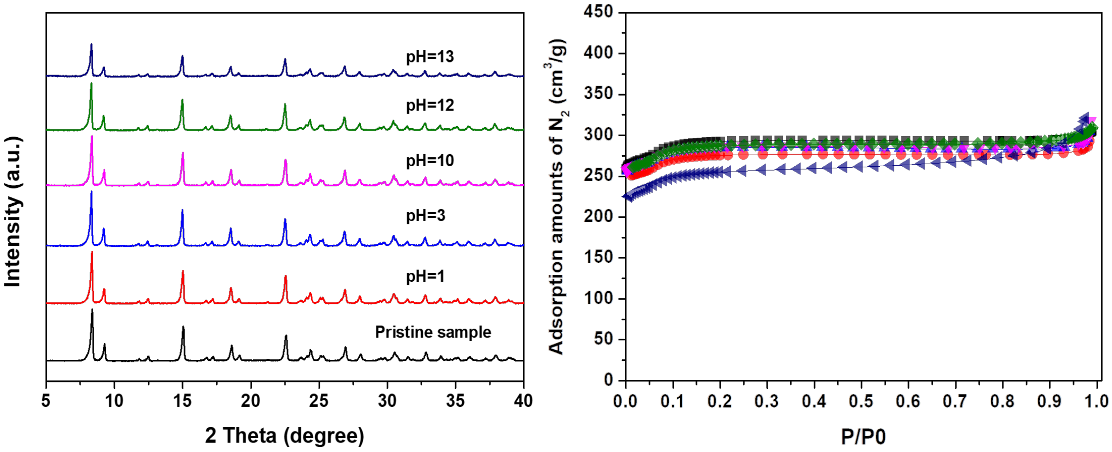

Supplementary Figure 11. PXRD patterns (left) and nitrogen adsorption isotherms at -196 °C (right) for KMF-1 before (■) and after treatment in various pH aqueous solutions pH 1 (●), pH 3 (▲), pH 10 (▼), pH 12 (◆), and pH 13(◀) at 25 °C for 24 h under stirring.

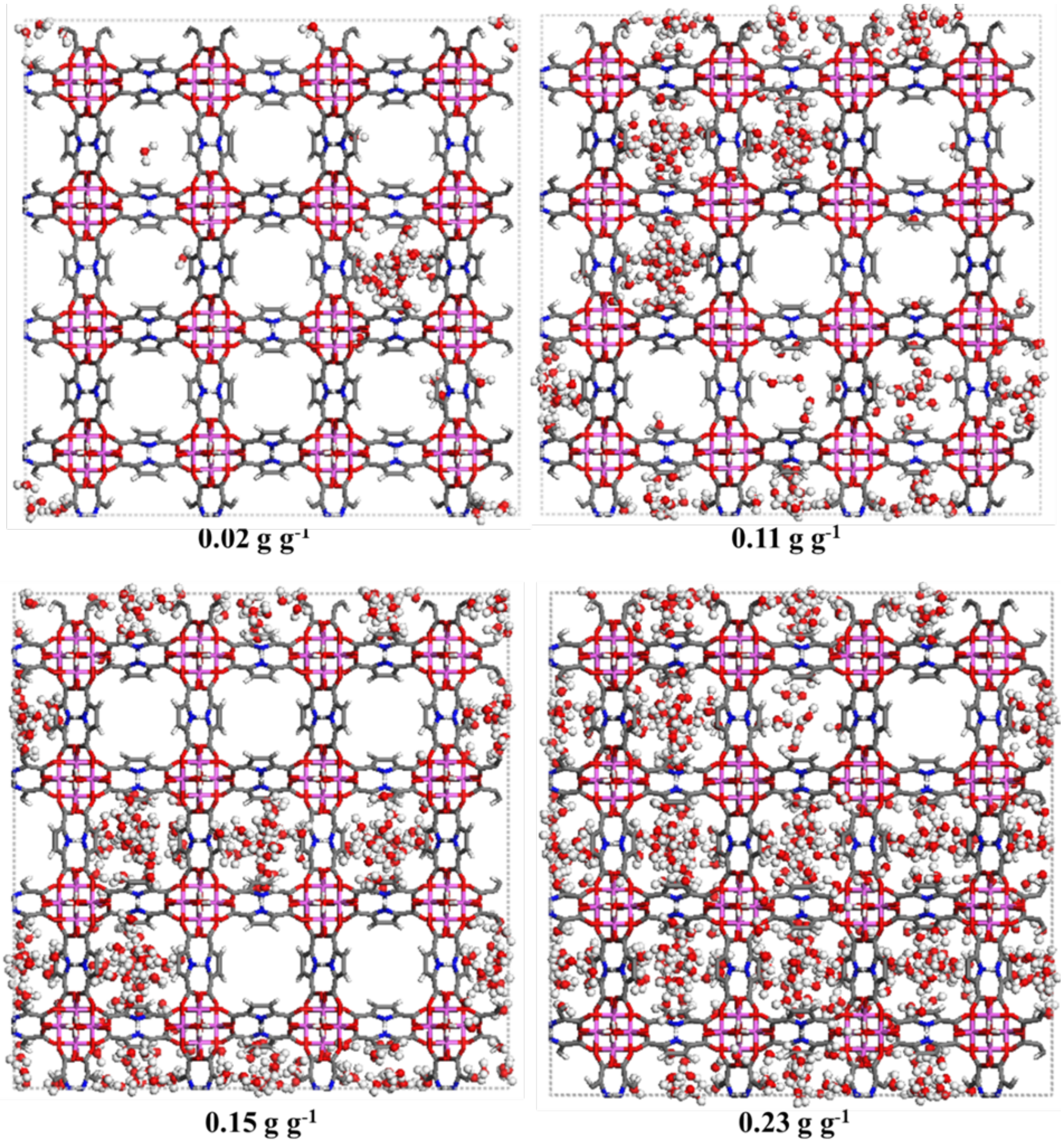

Supplementary Figure 12. Top view of KMF-1 and adsorbed water molecules corresponding to a water loading of 0.02 g g^{-1} (left-up), 0.11 g g^{-1} (right-up), 0.15 g g^{-1} (left-down) and 0.23 g g^{-1} (right-down). The water molecules are drawn with ball and stick representation, while the framework in stick representation. The color code for all atoms is the same than that employed in the main text.

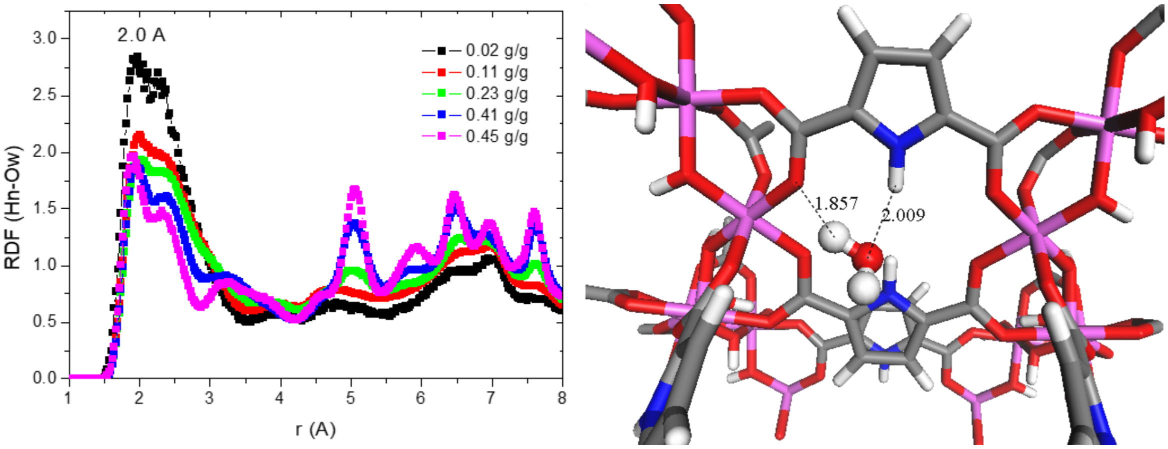

Supplementary Figure 13. (left) Radial distribution functions between the oxygen atoms of H_2O and the hydrogen atoms bounded to the –N atom of the pyrrole of the KFM-1, (right) illustrative snapshot showing the corresponding interactions.

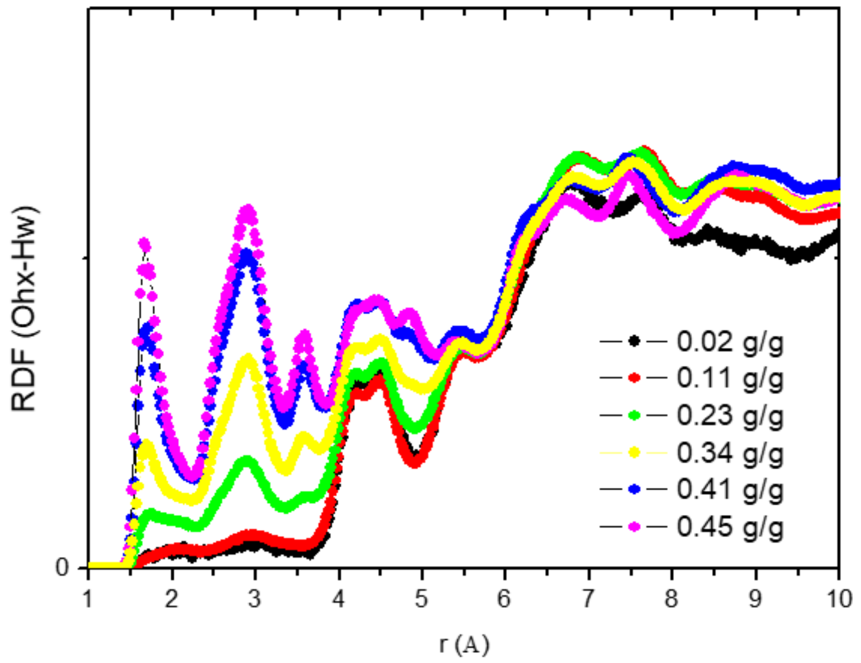

Supplementary Figure 14. Radial distribution functions between the hydrogen atoms of H_2O and the oxygen atoms of the hydroxyl group O(μ-OH) of KMF-1.

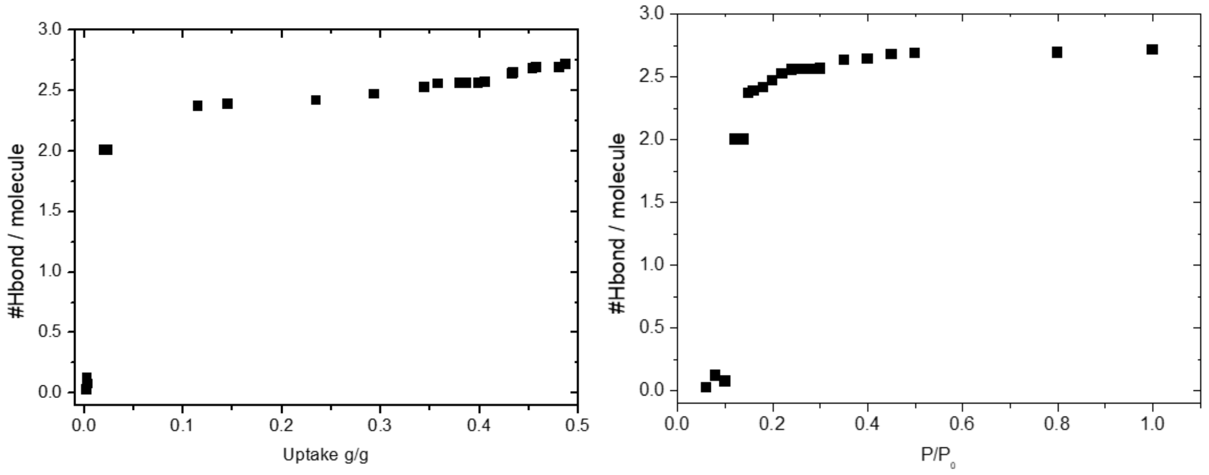

Supplementary Figure 15. Average number of hydrogen-bond (HB) per water molecule as a function of the water uptake (left) and pressure (right). Two water molecules are H-bonded if Ow-Ow distance is < 3.5 Å and the angle formed between the intramolecular O−H vector and the intermolecular O−O vector is shorter than 30°. At saturation, the average number of HB is 2.7 per H_2O molecule.

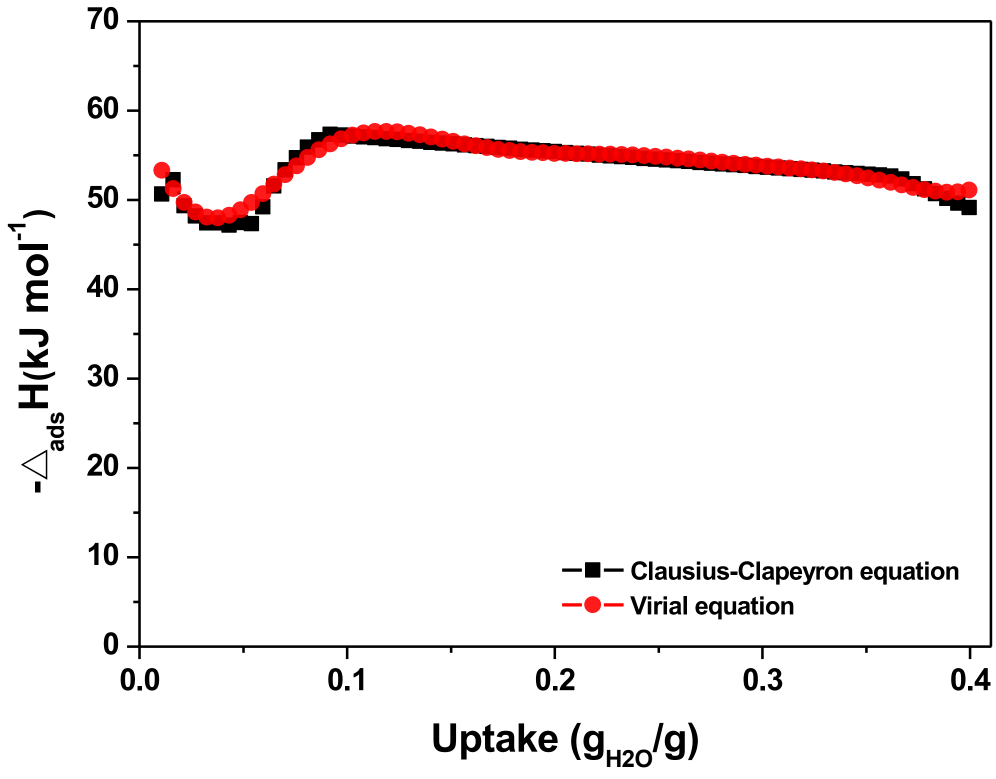

Supplementary Figure 16. Isosteric heat of adsorption as a function of water uptake for KMF-1.

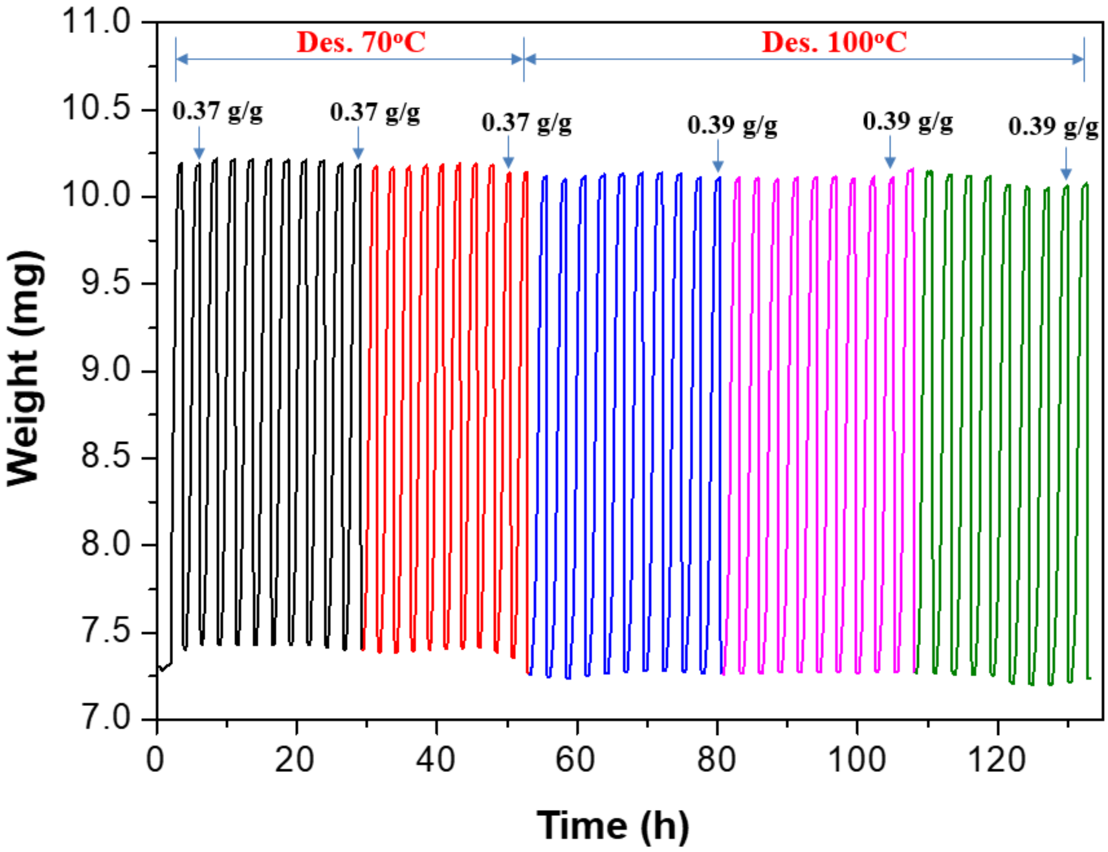

Supplementary Figure 17. Thermogravimetric analysis profile for 50 water adsorption-desorption cycles of KMF-1. Test conditions: adsorption at 30 °C in humid nitrogen (35% RH) and desorption at 70 °C in nitrogen with low humidity (4.8% RH) for 20 cycles followed by desorption at 100 °C in nitrogen with low humidity (1.4% RH) for 30 cycles. Prior to the multiple cycle experiment, the first cycle was carried out by a different condition: KMF-1 is dehydrated at 150 °C for 1 h in dry N_2, hydrated at 30 °C in humid nitrogen (35% RH).

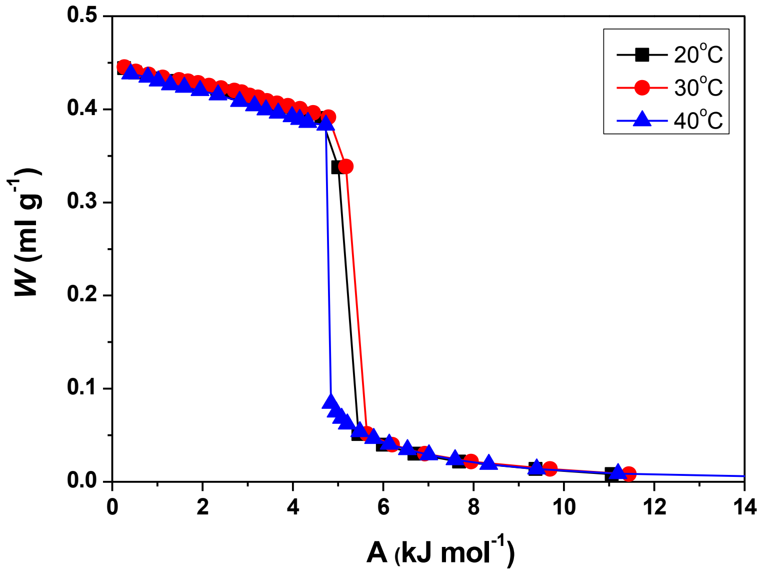

Supplementary Figure 18. Characteristic curves for water on KMF-1 at different temperatures.

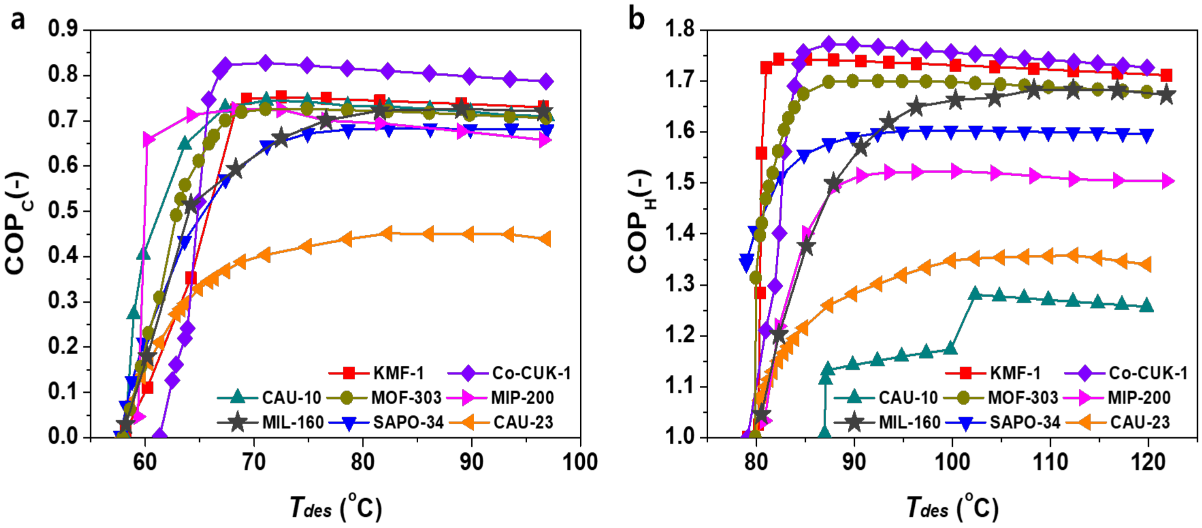

Supplementary Figure 19. Water sorption performances of KMF-1 in comparison with other adsorbents. **a**, Coefficient of Performance (COP$_C$) plots for AC conditions (T_{ev} = 5 °C, T_{ads} = 30 °C) as a function of desorption temperature (T_{des}). **b**, Coefficient of Performance (COP$_H$) plots for heat pump conditions (T_{ev} = 15 °C, T_{con}=30 °C, and T_{ads} = 45 °C) as a function of desorption temperature (T_{des}).

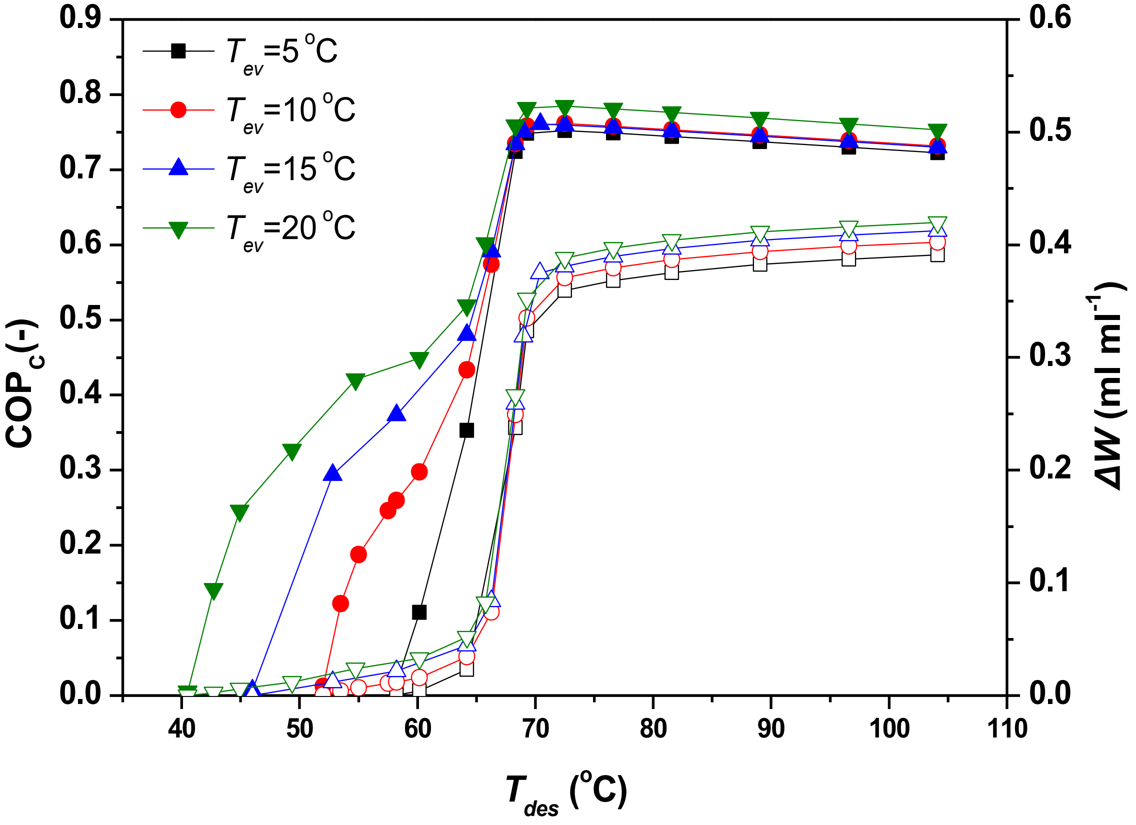

Supplementary Figure 20. Coefficient of Performance (COP$_C$) plots (*Closed symbol*) and working capacity based on absorbent volume (*Open symbol*) of KMF-1 at fixed adsorption temperature (T_{ads} = 30 °C) and condensation temperature (T_{con}=30 °C) as function of desorption temperatures (T_{des}= 40-105 °C) and evaporation temperatures (T_{ev}=5-20 °C)

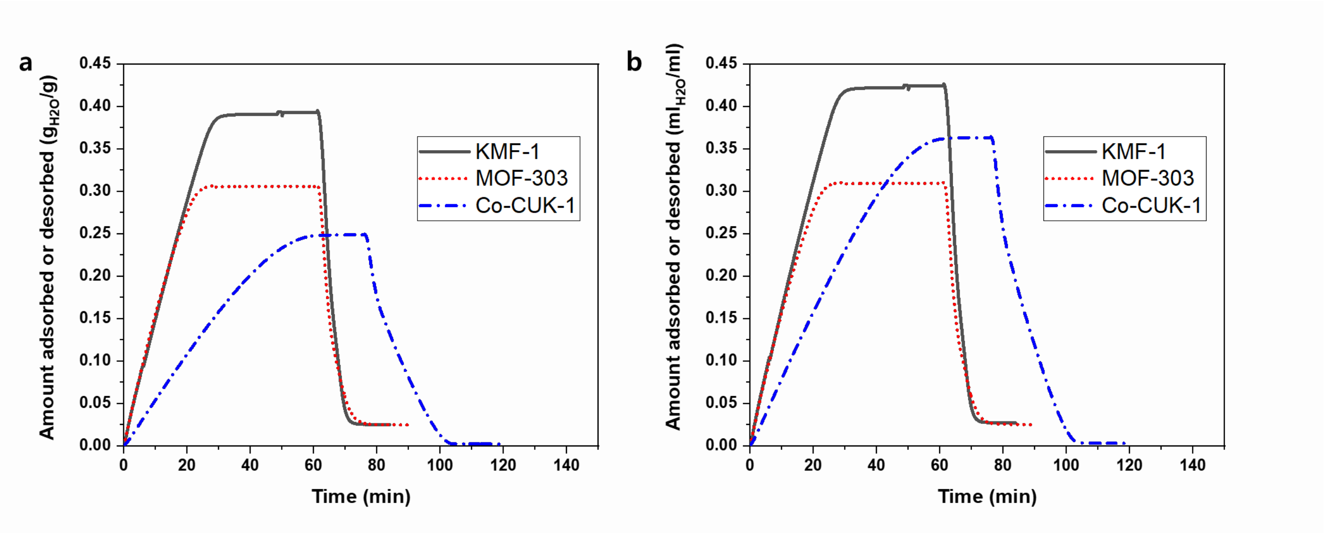

Supplementary Figure 21. (a) Gravimetric and (b) volumetric adsorption-desorption profiles of KMF-1, Co-CUK-1, and MOF-303. These profiles were plotted from the second adsorption-desorption cycle as a function of time, measured by TGA: adsorption at 30 ^oC and RH 35 % and desorption at 63 ^oC and RH 10 % in a nitrogen flow (100 ml/min). The second cycle profiles were obtained just after the first profiles of fully dehydrated adsorbents for adsorption at 30 ^oC and RH 35 %, followed by desorption at 63 ^oC and RH 10 %. The sample weight was determined by full dehydration at 150 ^oC under a dry nitrogen flow (100 ml/min). The ramping rate of desorption temperature is 20 ^oC/min.

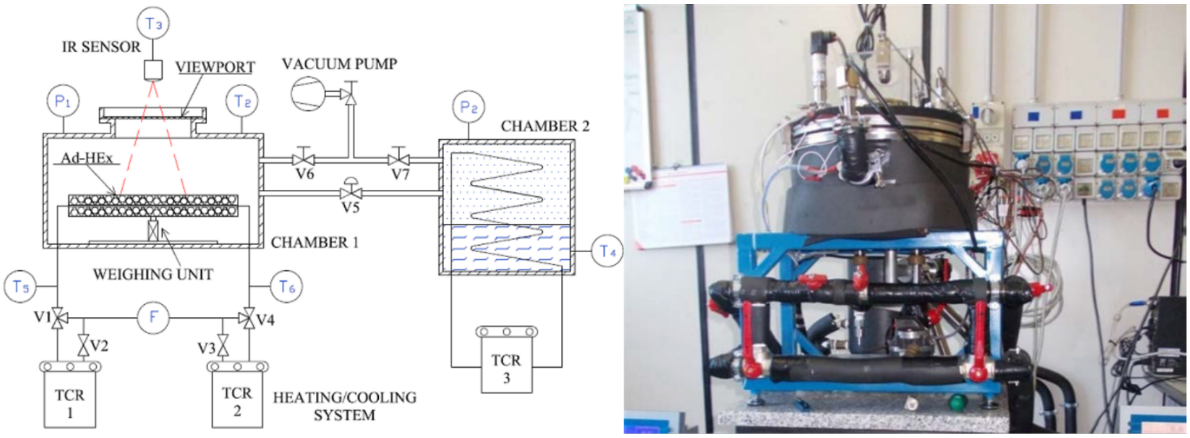

Supplementary Figure 22. View and scheme of the test facility (G-LTJ apparatus) installed at CNR-ITAE labs.

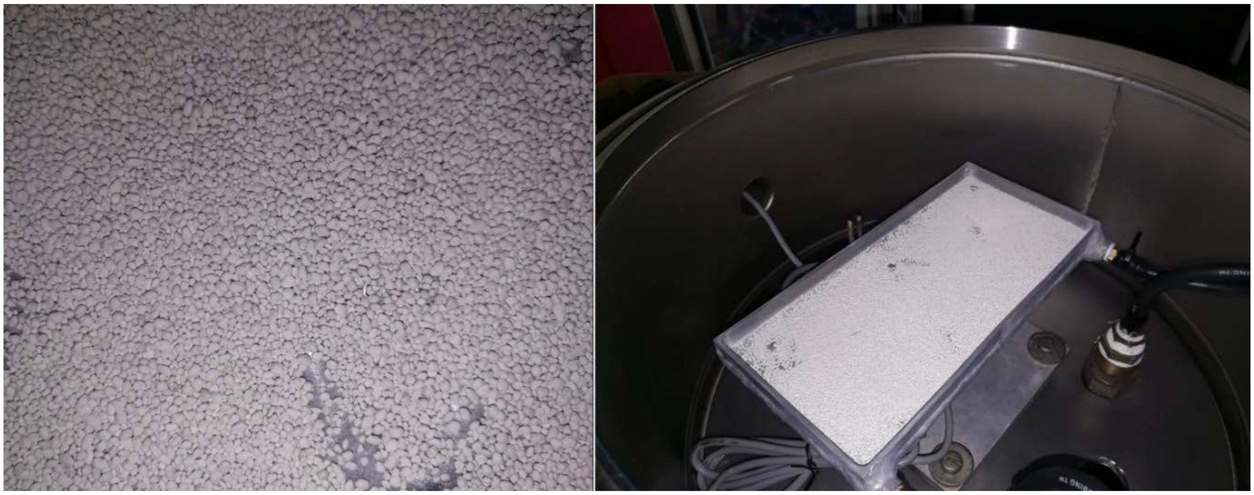

Supplementary Figure 23. Views of the tested adsorber.

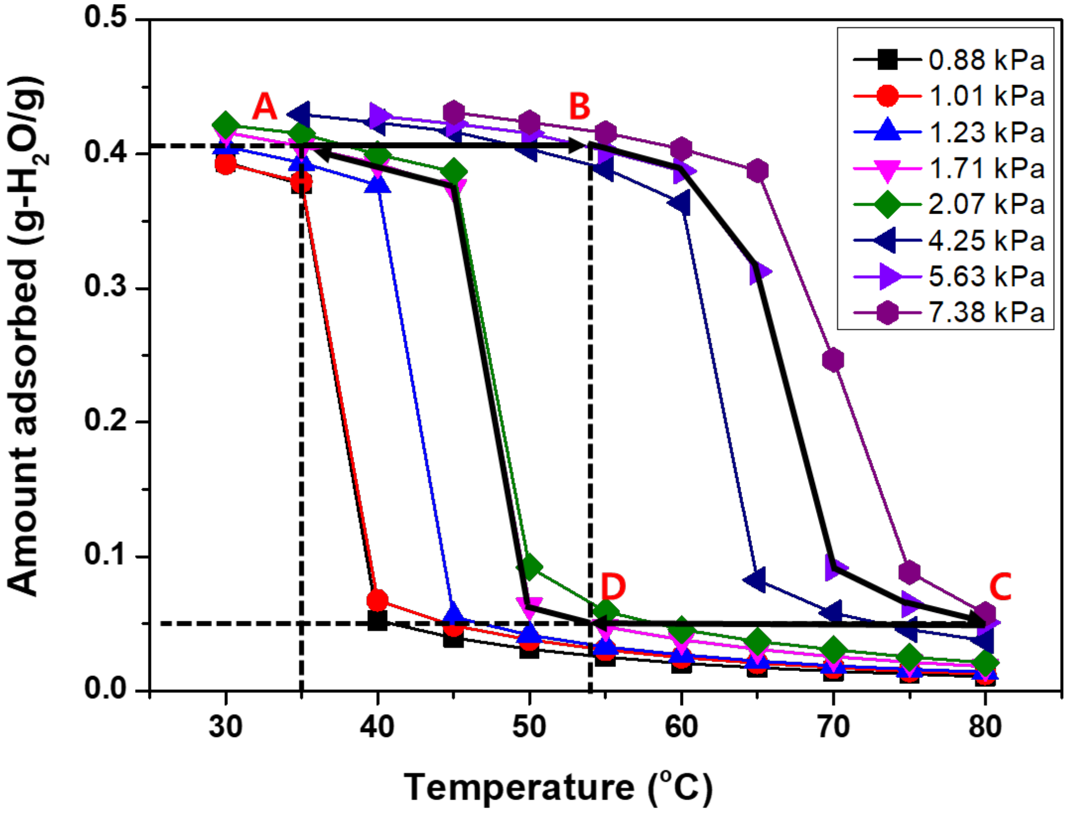

Supplementary Figure 24. Adsorption cooling cycle (T_{ev}=15 °C, T_{con}=35 °C, T_{des}=80 °C) plotted on the KMF-1 water sorption isobaric chart. The cycle consists of four steps: two for adsorption and two for desorption, in the order, isosteric cooling (D-C), isobaric adsorption (C-A), isosteric heating (A-B) and isobaric desorption (B-D).

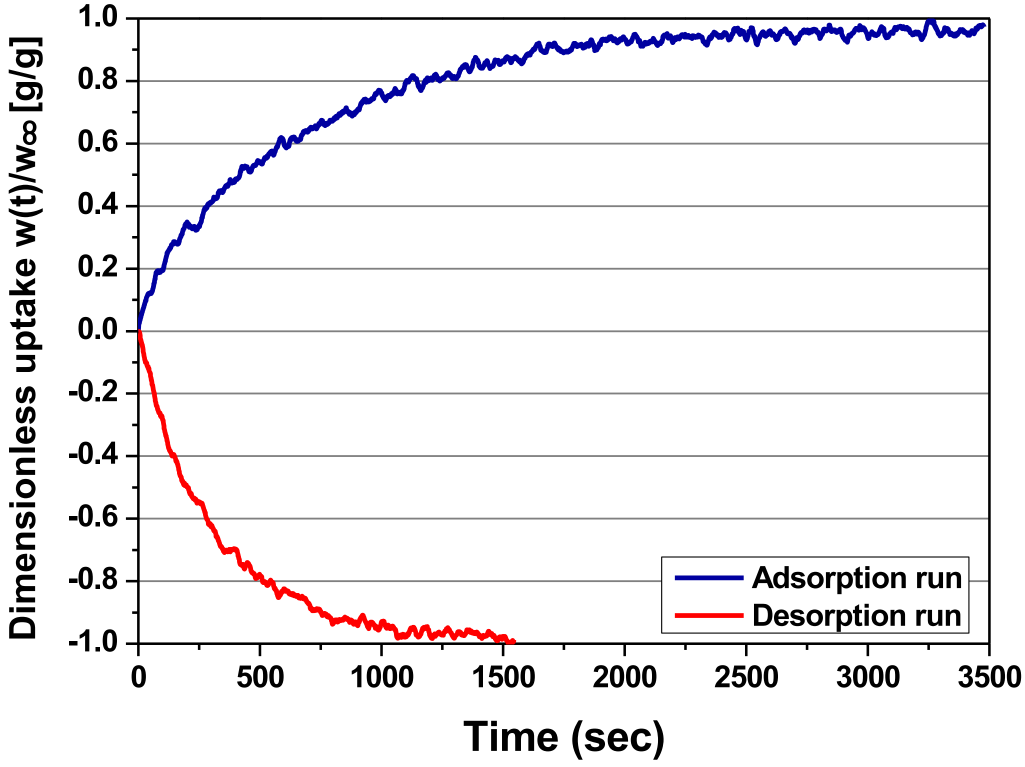

Supplementary Figure 25. Dimensionless uptake curves during adsorption and desorption runs under the operating conditions (T_{ev}=5 °C, T_{con}=30 °C, and T_{des}=70 °C).

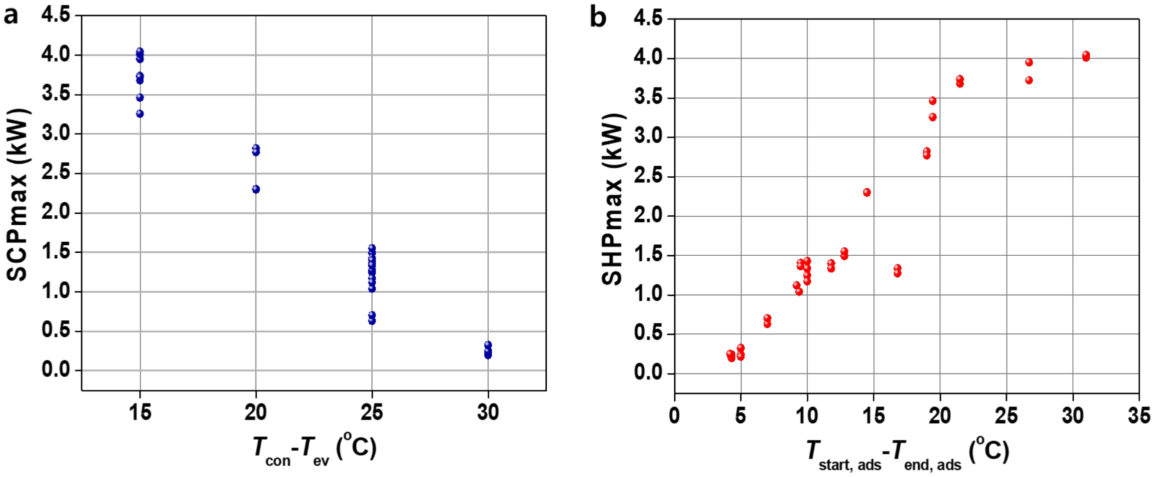

Supplementary Figure 26. Effect of temperature difference on SCP_{max}. **a**, Variation of SCP_{max} according to difference of T_{con} - T_{ev}. **b,** variation of SCP_{max} value according to difference $T_{start,\ ads}$ - $T_{end,\ ads}$.

Supplementary Table 1. Comparison of synthesis conditions and product yields of selected adsorbents

MOF	Reactant mole ratio	Reaction Temp. (^oC)	Reaction time (h)	Yield (%)	Space-time to Yield ($kg/m^3/day$)	Reference
KMF-1	1 $Al_2(SO_4)_3$: 1 PyDC: 2.5 NaOH	~ 120 (reflux)	12 h	93	68	This work
Co-CUK-1	3 $CoCl_2$: 2 PDC: 3 KOH	200	15 h	67	24	1
MIP-200	3.74 $ZrCl_4$: 1.25 H_4mdip: 663FA: 370 AA	120	48-72 h	96	7.2	2
CAU-10	1.5 $Al_2(SO_4)_3$: 1 $NaAlO_2$: 4 IPA: 8 NaOH	Reflux	15 h	95	93	4
CAU-23	3 $AlCl_3$: 1 $NaAlO_2$: 4 TDC: 8 NaOH	Reflux	4 h	84	137	5
MIL-160	1Al(OH)$(CH_3COO)_2$: 1 FDCA	Reflux	24 h	93	185	6
MOF-303	1$AlCl_3$: 1PyrzDC : 1.5 NaOH	~100 (reflux)	24 h	35	4	7
MOF-801P	1$ZrOC_l2$: 1 FUA: 37 FA: 52 DMF	120	12 h	63	148	8

* Abbreviation of chemicals: PyDC (2,5-pyrroledicarboxylic acid); IPA (isophthalic acid); TDC (2,5-thiophenedicarboxylic acid); FDCA (2,5-furandicarboxylic acid); PyrzDC (3,5-pyrazoledicarboxylic acid); FUM (fumaric acid); FA (formic acid); DMF (N,N-dimethylformamide); AA (Acetic anhydride); PDC (2,4-pyridine dicarboxylic acid); H_4mdip (3,3’,5,5’-tetracarboxydiphenylmethane).

Supplementary Table 2. DFT-derived DDEC atomic partial charges for the KFM-1 framework

**Al1**	1.6740	**H3**	0.4256
**C1**	-0.1690	**N1**	-0.213401
**C2**	0.0344	**O1**	-1.004704
**C3**	0.6082	**O2**	-0.606565
**H1**	0.1372	**O3**	-0.582
**H2**	0.2993		

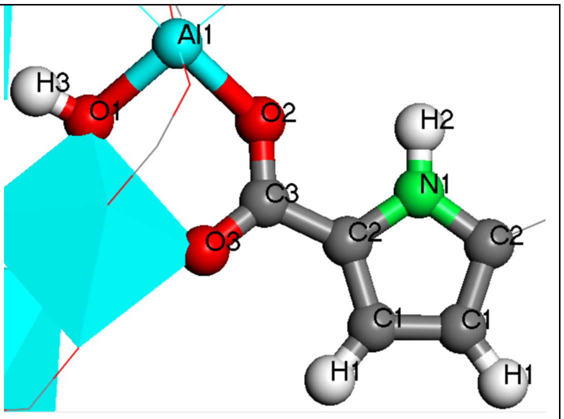

Supplementary Table 3. Elemental Analysis of KMF-1

	C weight fraction (wt %)	N weight fraction (wt %)	H weight fraction (wt %)	Defined chemical formula
Calculated	35.78	6.96	2.22	$Al(OH)(PyDC)(H_2O)_{0.23}$
Observed	33.27	6.64	2.22	$Al(OH)(PyDC)_{0.93}(H_2O)_{0.23}$

Supplementary Table 4. Experimental data on Rietveld refinement of anhydrous KMF-1

Material	KMF-1 hydrated	KMF-1 dehydrated
Unit cell Composition	C_{96} Al_{16} N_{16} O_{152}	C_{96} Al_{16} N_{16} O_{80}
Refined structure		
Symmetry	Tetragonal	Tetragnoal
space group	$I4_1md$	$I4_1/amd$
a, Å	21.1772(2)	21.225(2)
c, Å	10.70115(17)	10.6424(16)
cell volume, $Å^3$	4799.18(10)	4794.5(10)
Wavelength/Å	1.5225	0.9000
Radiation type	Synchrotron Radiation	Synchrotron Radiation
No. Observation	1134	1071
No. Parameters	74	44
No. Restraints	0	0
No. Constraints	2	1
Max Change/s.u.	0.0389	0.0342
Largest Peak	0.59	0.49
Deepest Hole	-0.57	-0.62
$R_{p,}$ %	8.21	4.09
$R_{wp,}$ %	10.71	5.97
$R_{F,}$ %	7.90	7.62
GOF	0.52	1.02

Supplementary Table 5. Comparison of BET areas and pore volumes for KMF-1 before and after hydrothermal and acid-base treatments.

Sample	BET area, S_{BET} (m^2 g^{-1})	Total pore volume, V_p (cm^3 g^{-1})
Pristine KMF-1	1130	0.472
After hydrothermal treatment	1090	0.438
After pH 1treatment	1060	0.452
After pH 3 treatment	1100	0.451
After pH 10 treatment	1100	0.451
After pH 12 treatment	1100	0.477
After pH 13 treatment	980	0.505

Supplementary Table 6. Water sorption properties and energy storage capacities of water adsorbents

Material	Crystal density (g/cm^3)	Working capacity[a]		Specific energy capacity[b]		Energy storage capacity[c]	
		(g·g^{-1})	(ml·ml^{-1})	(Wh·kg^{-1})	(kWh·m^{-3})	(Wh·kg^{-1})	(kWh·m^{-3})
KMF-1	1.080	0.33	0.36	243.7 (246.3)	263.2 (266)	323	348
MIP-200	1.16	0.14[d]	0.16[d]	88.4 (150.2)[d]	102.5 (174.2)[d]	189[d]	220[d]
Co-CUK-1	1.46	0.24[d]	0.35[d]	173.6 (180.7)[d]	253.4 (263.8)[d]	207[d]	302[d]
MOF-303	1.012	0.254[d]	0.257[d]	178.3 (188.0)[d]	180.4 (190.2)[d]	255[d]	258[d]
MOF-801	1.59	0.10[d]	0.16[d]	38.0 (56.4)[d]	60.4 (89.7)[d]	103[d]	164[d]
MIL-160	1.068	0.11[d]	0.12[d]	67.7 (74.7)[d]	72.3 (79.8)[d]	108[d]	115[d]
CAU-10	1.15	0.26[d]	0.30[d]	185.3 (189)[d]	213 (217.4)[d]	238[d]	274[d]
CAU-23	1.07	0.016[d]	0.017[d]	8.23 (136.6)[d]	8.82 (146.2)[d]	168[d]	180[d]
MIL-125-NH_2	0.80	0.049[d]	0.039[d]	33.9 (252.1)[d]	27.1 (201.1)[d]	310[d]	248[d]
SAPO-34	1.43	0.10[d]	0.14[d]	115 (120)[d]	165 (172)[d]	115[d]	165[d]

[a]Working capacity deduced from one refrigeration cycle at T_{ev} = 5 °C, T_{ad} = 30 °C, T_{con} = 30 °C, and T_{des} =70 °C;

[b]Specific energy capacity of material in one refrigeration cycle at T_{ev} = 5 °C, T_{ad} = 30 °C, T_{con} = 30 °C, and T_{des} =70 °C (T_{ev} = 10 °C for values in parentheses);

[c]Energy storage capacity per unit weight or volume of adsorbent at T_{ev} = 10 °C, T_{ad} = 30 °C, T_{con} = 30 °C, and T_{des} = 70 °C.

[d]Values of working capacity, heat from evaporator, and heat storage capacity for Co-CUK-1, MIP-200, MOF-303, MIL-160, CAU-10, CAU-23, MIL-125-NH_2, SAPO-34 and MOF-801 were calculated by characteristic curves and data taken from references[1-3,5,7-9]

Supplementary Table 7. Main features of the tested adsorber

Surface area of KMF-1 granule, m^2/g	1040
Sorbent dry mass, kg	0.0127
Sorbent grain size, mm	0.5-1.5
Hex dimension L×W, mm	200×100
Total HT area, m^2	0.02
HT area/dry mass (S/m), m^2/kg	1.575

Supplementary Table 8. Operating conditions of tests

Evaporation temperature, T_{ev} [°C]	5/10/15
Condensation temperature, T_{con} [°C]	30/35/40
Regeneration temperature, T_{des} [°C]	65/70/75/80

Supplementary Table 9. Temperature drops for the adsorption steps under selected operating conditions

Test condition ($T_{des}/T_{con}/T_{ev}$)	Type	T drop/jump	T_e (Psat)	Delta Uptake [wt.%]
65°C/30°C/5°C	adsorption	39.4-30 °C	5 °C (0.88 kPa)	30.82 %
70°C/30°C/5°C	adsorption	40-30 °C	5 °C (0.88 kPa)	33.6 %
80°C/30°C/5°C	adsorption	46.8-30 °C	5 °C (0.88 kPa)	35.4 %
70°C/35°C/10°C	adsorption	44.2-35 °C	10 °C (1.23 kPa)	30%
75°C/35°C/10°C	adsorption	45-35 °C	10 °C (1.23 kPa)	33.2%
80°C/35°C/10°C	adsorption	46.8-35 °C	10 °C (1.23 kPa)	34.2%
75°C/40°C/10°C	adsorption	44.2-40 °C	10 °C (1.23 kPa)	28.9%
80°C/40°C/10°C	adsorption	45-40 °C	10 °C (1.23 kPa)	31.9%
65°C/30°C/15°C	adsorption	49.45-30 °C	15 °C (1.71 kPa)	33.3 %
70°C/30°C/15°C	adsorption	51.5-30 °C	15 °C (1.71 kPa)	35.5%
75°C/30°C/15°C	adsorption	56.7-30 °C	15 °C (1.71 kPa)	37%
80°C/30°C/15°C	adsorption	61-30 °C	15 °C (1.71 kPa)	37.85%
70°C/35°C/15°C	adsorption	49.5-35 °C	15 °C (1.71 kPa)	31.3%
80°C/35°C/15°C	adsorption	54-35 °C	15 °C (1.71 kPa)	35.5%
70°C/40°C/15°C	adsorption	47-40 °C	15 °C (1.71 kPa)	14.3%
75°C/40°C/15°C	adsorption	49.5-40 °C	15 °C (1.71 kPa)	30.4%
80°C/40°C/15°C	adsorption	52.8-40 °C	15 °C (1.71 kPa)	33.6%

Supplementary Table 10. Specific cooling power (SCP) values and sorption characteristic times of shaped KMF-1 obtained from kinetic water sorption measurements under several operating conditions*

Cycle	Operating temp. (^oC)			Kinetic evaluation results				
	T_{ev}	T_{con}	T_{des}	$t_{80\%}$ (sec)	$\tau_{80\%}$ (sec)	Δw_{∞} (g g^{-1})	$SCP_{80\%}$ (kW kg^{-1})	SCP_{max} (kW kg^{-1})
1	5	30	65	1173	738	0.31	0.52	1.04
2	10	35	70	1073	663	0.30	0.55	1.12
3	15	30	70	379	238	0.33	1.60	3.74
4	15	35	80	509	311	0.35	1.38	2.82
5	15	40	75	833	533	0.30	0.72	1.40

*Definition: $t_{80\%}$; required time for 80 % water uptakes of theoretical maximum water uptakes at given condition, $\tau_{80\%}$; characteristic sorption time fitted by interpolation method with kinetic curve of 80% water uptakes. $SCP_{80\%}$; Specific cooling power at $t_{80\%}$, SCP_{max} ; theoretical maximum cooling power.

Supplementary Table 11. Specific heating power (SHP) values sorption characteristic times of shaped KMF-1 obtained from kinetic water sorption measurements under various operating conditions*

Cycle	Operating temp. (^{o}C)			Kinetic evaluation results				
	T_{ev}	T_{con}	T_{des}	$t_{80\%}$ (sec)	$\tau_{80\%}$ (sec)	Δw_{∞} (g g^{-1})	$SHP_{80\%}$ (kW kg^{-1})	SHP_{max} (kW kg^{-1})
1	15	35	75	436	264	0.34	1.50	3.11
2	15	35	80	257	161	0.35	2.68	5.34
3	15	40	80	433	257	0.34	1.49	3.15
4	15	40	85	299	174	0.36	2.31	4.96
5	15	45	85	443	303	0.31	1.32	2.81
6	5	35	80	356	229	0.32	1.77	3.37

*Definition: $t_{80\%}$; required time for 80 % water uptakes of theoretical maximum water uptakes at given condition, $\tau_{80\%}$; characteristic sorption time fitted by interpolation method with kinetic curve of 80% water uptakes. $SHP_{80\%}$; Specific heatng power at $t_{80\%}$, SHP_{max} ; theoretical maximum heating power.
